# Social Inequalities in Height: Persisting Differences Today Depend upon Height of the Parents

**DOI:** 10.1371/journal.pone.0029118

**Published:** 2012-01-06

**Authors:** Bruna Galobardes, Valerie A. McCormack, Peter McCarron, Laura D. Howe, John Lynch, Debbie A. Lawlor, George Davey Smith

**Affiliations:** 1 School of Social and Community Medicine, Causal Analysis in Translation Epidemiology Centre, University of Bristol, Bristol, United Kingdom; 2 Section of Environment and Radiation, International Agency for Research on Cancer, Lyon, France; 3 St John of God Hospital, Stillorgan, Ireland; 4 School of Population Health and Clinical Practice, University of Adelaide, Adelaide, Australia; Uniformed Services University of Health Sciences, United States of America

## Abstract

**Background:**

Substantial increases in height have occurred concurrently with economic development in most populations during the last century. In high-income countries, environmental exposures that can limit genetic growth potential appear to have lessened, and variation in height by socioeconomic position may have diminished. The objective of this study is to investigate inequalities in height in a cohort of children born in the early 1990s in England, and to evaluate which factors might explain any identified inequalities.

**Methods and Findings:**

12,830 children from The Avon Longitudinal Study of Parents and Children (ALSPAC), a population based cohort from birth to about 11.5 years of age, were used in this analysis. Gender- and age-specific z-scores of height at different ages were used as outcome variables. Multilevel models were used to take into account the repeated measures of height and to analyze gender- and age-specific relative changes in height from birth to 11.5 years. Maternal education was the main exposure variable used to examine socioeconomic inequalities. The roles of parental and family characteristics in explaining any observed differences between maternal education and child height were investigated.

Children whose mothers had the highest education compared to those with none or a basic level of education, were 0.39 cm longer at birth (95% CI: 0.30 to 0.48). These differences persisted and at 11.5 years the height difference was 1.4 cm (95% CI: 1.07 to 1.74). Several other factors were related to offspring height, but few changed the relationship with maternal education. The one exception was mid-parental height, which fully accounted for the maternal educational differences in offspring height.

**Conclusions:**

In a cohort of children born in the 1990s, mothers with higher education gave birth to taller boys and girls. Although height differences were small they persisted throughout childhood. Maternal and paternal height fully explained these differences.

## Introduction

Height is a highly heritable trait [1]. Nevertheless, there is substantial variation of adult height, both in populations from different countries [2], and within a population over time, as the range in growth rates, from 10 to 30 mm/decade, across European populations demonstrate [3]. This variability strongly suggests that environmental, and hence, potentially modifiable factors, have a role in determining height [4]. These positive secular trends have been attributed to improvements in health, and economic, and social conditions during childhood.

Attained adult height is determined by the potential of a child's genotype and the restrictions that the environment places on this [5]. Environmental influences acting in early life, a period of rapid growth and development, are particularly important [6]–[9]. Thus, socioeconomic circumstances, overcrowding and childhood illnesses [10], dietary supplementation [11], maternal smoking during pregnancy and parental smoking in childhood [12]–[14] are all related to variations in infant and childhood height, and hence to attained adult height [15], [16]. These exposures are potentially modifiable and are differently distributed across socioeconomic groups. On the other hand, recent increases in average population height in high income countries have led some authors to suggest that differences in height are now only minimally influenced by environmental factors in these countries [17]. However, we have previously reported a small but clear gradient in birth length, which persists throughout childhood to mean age 10 years, across levels of maternal education in the ALSPAC cohort, a birth cohort of children born in the 1990s in the UK [18], [19]. In this paper we extend these earlier findings by exploring the role of potentially modifiable characteristics which might explain height differences. This is important because height is related to future health (including cardiovascular disease and cancer) and wellbeing [20]–[22], and understanding the mechanisms that drive socioeconomic inequalities in height growth in childhood might provide means for interventions that could prevent these and related inequalities in health and wellbeing.

Thus, the aim of this study is to better understand what drives socioeconomic differentials in height from birth to childhood in a contemporary population of UK children born in the early 1990s.

## Methods

### Study design

The Avon Longitudinal Study of Parents and Children (ALSPAC) is a population-based study investigating environmental and genetic factors that affect health and development of children. The study methods are described in detail elsewhere [23] (http://www.alspac.bris.ac.uk). Briefly, pregnant women living in three health districts in Bristol, England, who had an expected date of delivery between 1 April 1991 and 31 December 1992, were eligible. The recruited ALSPAC sample consists of 14,541 pregnancies that resulted in 14,676 known foetuses.

Detailed data about her socioeconomic background, health, welfare and lifestyle characteristics were obtained from the mother using four self-reported questionnaires throughout the pregnancy. Data on delivery and birth measurements were obtained by ALSPAC staff or were otherwise extracted from medical records. Since delivery, regular questionnaires have been completed by the child's main caregiver (most commonly their mother) and as they became older, the children themselves.

Ethical approval for the study was obtained from the ALSPAC Law and Ethics Committee and the Local Research Ethics Committees. Written informed consent was obtained from all participants involved in the study.

### Variable description

Maternal education was ascertained from the antenatal 32-week questionnaire. Education was coded using an ascending mutually exclusive five point scale of highest educational achievement: “None/Certificate of Secondary Education (CSE)”, 2: “Vocational”, 3: “Ordinary- (O-) level (exams taken usually at age 16 years at the completion of legally required school attendance and equivalent to the present UK General Certificate of Secondary Education (GCSE))”, 4: “Advanced- (A-) level (exams taken usually at age 18 years”, and 5: “University Degree”. Levels 1 to 3 refer to different levels (from lowest to highest) of educational qualifications most commonly attained at 16 years of age (the minimum age at which someone could legally leave education in the UK at the time that these mothers were in school); level 4 refers to educational qualifications gained at 18 years of age. Mothers with no educational qualifications most often left the question unanswered which was recoded to none, and those who responded ‘not known’ were left as missing. A previous report on this cohort found similar socioeconomic differentials in birth length and childhood growth irrespective of whether maternal education, head of household occupational social class or father's education was used as the measure of socioeconomic position (SEP) [18]. Therefore, maternal education was chosen as indicator of SEP in these analyses. Maternal height was obtained from self-report in one of the antenatal questionnaires (for 90% of the mothers) or from the first post-natal questionnaire (10%). A food frequency questionnaire administered at week 32 of pregnancy recorded maternal diet during gestation. This was converted to total energy (Kcal/day), protein, total fat, saturated fat, polyunsaturated fat, monounsaturated fat and carbohydrate intake (all in grams/day). Self-reported maternal smoking during the 1^st^, 2^nd^ and 3^rd^ trimesters was measured. A variable categorized as “Never” or “Ever” smoking during pregnancy and a variable indicating the number of trimesters the mother smoked were created. Finally, the number of previous pregnancies (both live births and stillbirths) and number of living children were reported by the mother.

The height, weight, and smoking habit during pregnancy of the mother's partner were obtained from a partner's self-completed questionnaire that was passed to them via the mother. For 95.5% of the children the mother's partner was the biological father (by mother's self-report). Mid-parental height was calculated using both parents' height adapting Galton's formula [24], [25] to the ALSPAC population (Appendix S1).

Data on delivery and birth measures (crown-heel length and head circumference) were obtained by trained staff of the ALSPAC team for babies born in the two major maternity hospitals in the region and from medical records for the other participants. Gestational age was estimated using the mother's last menstrual period in most cases and through obstetric assessment for the rest. Whether the mother breastfed, and duration of breastfeeding, were ascertained at 6 months and categorized into a composite variable, as “Never or up to one month” versus “more than one month”.

Height after birth was measured by health visitors and general practitioners as part of standard childcare in the UK. The examinations take place at around the 8^th^ week (median: 8 weeks, range: 1.3 to 58.6 weeks), 8^th^ month (median: 9 months, range: 1.2 to 21 months), 18^th^ month (median: 18 months, range: 10 to 30 months) and at the pre-school child visit at 3.5 years (median: 3.6 years, range: 2.5 to 5.9 years). Thereafter, the whole cohort of children was invited to attend clinical examinations. The first ALSPAC direct measurement of height occurred at an average of 7.5 years (range: 6.8 to 9.2 years) and four subsequent yearly examinations were held at ages 8.5 years (range: 7.5 to 10.5 years), 9.5 years (range: 8.7 to 11.7 years), 10.5 years (range: 9.8 to 11.3 years) and 11.5 years (range: 10.4 to 13.6 years). There are in total a maximum of 10 measurements of height per child. In the clinics (from age 7.5 years) height was measured by trained technicians to the last complete millimetre using the Harpenden stadiometer (Holtain Ltd). As far as possible, all children were measured in their underclothes with their shoes removed. For all measurements taken, the tester recorded any problems that may have affected accuracy. In a previous study we have shown that heights assessed from birth to pre-school by health visitors were accurate, by comparing these with research clinic measurements completed on a random 10% sub-sample of the ALSPAC cohort [26].

### Statistical analysis

Gender- and age-specific z-scores for length/height were calculated. Z-scores control for the association of age and gender on height and its change, and standardise for the increasing variance of the measurements with increasing age. As there was considerable variation in the ages at which the children had their measurements taken, z-scores were calculated within the following time intervals, irrespective of the visit when they were obtained: i) for birth length, gestational age in 1 week intervals for those born from 37 to 43 weeks; ii) for length/height between the 1^st^ week and 6 months, child's age in 1 week intervals; and, iii) heights beyond 6 months, child's age in 1 month intervals. Time intervals with too few observations for appropriate calculation of a z-score were combined with the earlier interval. An alternative method to standardise height using a locally weighted smoother was evaluated but produced similar standardised values (results available from the authors). Z-scores were preferred as they are more easily interpreted and translated to the original scale.

Exploratory cross-sectional analyses were carried out at each visit to evaluate the association of mother's education with child's height at each age. Multilevel modelling was carried out to model height (in z-scores) change with age and to evaluate the role of the mother's educational level on the child's height z-score trajectory. There was strong statistical evidence in both boys and girls, that a random intercept and random slope model provided a better fit to the data than a model that included a random intercept only (maximum likelihood ratio test between a random intercept only and a random intercept and slope model p-value<0.001). The association with maternal education was evaluated as a categorical and ordinal variable and gender differences in the educational effect on height were tested with an interaction term.

The following explanatory variables were evaluated to test mechanisms that could explain differences in child's height according to mother's education. These variables were chosen based on previous reports of the literature of determinants of child's height. Maternal age at delivery (years), maternal height (cm) and body mass index (BMI in kg/cm^2^), number of children, gestational age (weeks) maternal smoking during gestation, child's early nutrition measured with breastfeeding and maternal food frequency questionnaire at 32-week pregnancy, paternal height (cm), BMI and smoking habit during gestation. These variables were added in the multilevel model as fixed effects using restricted maximum likelihood. Wald tests were used to evaluate the effect of adding each fixed term. All continuous variables were centred on their mean value. Models were also adjusted for a dummy variable indicating the visit at which the measurement took place to adjust for potential differences occurring between measurements.

All analyses were repeated excluding observations with z-scores of height above 2 or below −2 which allowed evaluation of the influence of extreme values on the model (by definition, around 5% of the data) to test the robustness of the results and the assumptions of the models and improve the normality distribution of the outcome variable. All analyses were carried out using STATA (version 10.1 for Windows).

## Results

The analyses were restricted to singletons as in-utero conditions may differ for multiple pregnancies (390 multiple pregnancies were excluded) and to pregnancies that resulted in a child alive at 27 days after birth (635 observations excluded, including all foetal losses at any stage of the pregnancy) and were term births (≥37 weeks) (693 observations excluded). Children who had no height measure available throughout the entire follow-up were excluded (n = 115). Finally, measurements of birth length that were obtained later than one week after delivery were not considered (180 measurements), as these may no longer reflect birth length, although all subsequent measures of these children were used in the analyses. The final sample included 12,830 children (6,579 boys with a median of 6 measurements (interquartile range (IQR): 4 to 9); 6,251 girls with a median of 7 measurements (IQR: 4 to 9).

**Figure 1:**
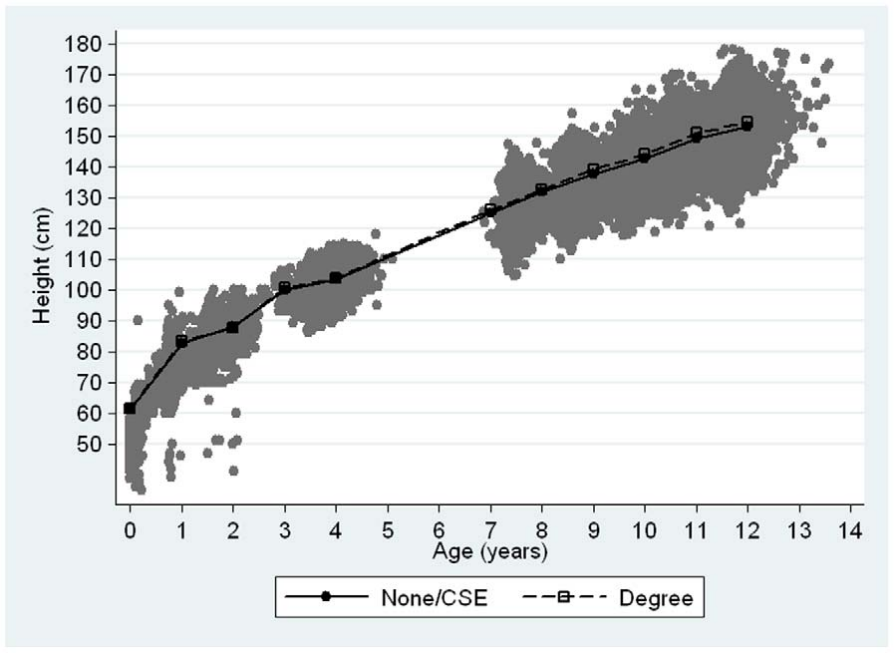
Scatter and mean height (cm) among children whose mothers had none or basic education (CSE) and those whose mothers had a degree*. * There were few children aged 5 or 6 and 13, thus their mean height was calculated jointly with those aged 4 and 12, respectively.

Mother's educational level was available for 89.4% of the children (n = 11,473). Almost 20% (n = 2,289) had either no education or education to CSE level, 9.8% had vocational education, 34.8% had O-levels, 22.5% had A-levels and 12.9% of the mothers had a university degree. The median number of height measurements was greater with higher maternal educational level: 4 measurements (IQR: 3 to 5) for mothers with no education or CSE level, 6 measurements (IQR: 4 to 9) for those with vocational training, 7 measurements (IQR: 5 to 9) for mothers with O-levels, and 8 measurements (IQR: 5 to 9) for mothers with A-level or university degrees. Mean height increased with age similarly among boys and girls. Boys tended to be slightly taller than girls up until the age of 7–8 years. Birth length and height were consistently higher with increasing levels of mother's education although the magnitude of these differences across educational groups was relatively small (Figure 1).

Table 1 shows the characteristics of the participants by maternal educational groups. With few exceptions (gestational age and some micronutrients from maternal diet during pregnancy), all characteristics investigated varied by maternal education. The social patterning of these characteristics was similar among boys and girls (all interaction p-values≥0.04).

**Table 1:** Maternal, child and partner's characteristics according to maternal educational level, adjusted for gender.

	None/CSE		Vocational		O –level		A-level		Degree		Trend p-value
	n	mean	n	mean	n	mean	n	mean	n	mean	
**Child characteristics**											
Gestational age (weeks)	2289	39.8	1128	39.7	3988	39.8	2583	39.7	1485	39.8	0.74
Breastfeeding >1month, n, %	1717	37.9	918	42.4	3524	56.2	2358	73.9	1402	87.9	<0.001
**Mother characteristics**											
Mother's age at delivery (years)	2289	26.9	1128	26.9	3988	27.5	2583	29.5	1485	31.4	<0.001
Number of children	2113	1.13	1068	0.84	3831	0.78	2486	0.74	1450	0.68	<0.001
Maternal height (cm)	2007	162.8	1042	163.2	3766	164.1	2469	164.4	1432	165.8	<0.001
Maternal BMI (kg/m^2^)	1855	23.5	967	23.3	3575	23.0	2359	22.7	1383	22.1	<0.001
Maternal diet pregnancy at 33 weeks											
Energy (kcal/day)	2151	1739.9	1080	1751.0	3858	1774.4	2502	1781.7	1435	1828.8	<0.001
Protein intake (g/day)	2151	60.3	1080	62.9	3858	66.1	2502	68.6	1435	71.7	<0.001
Total fat (g/day)	2151	69.3	1080	70.3	3858	70.7	2502	69.7	1435	70.5	0.26
Saturated fat (g/day)	2151	29.4	1080	29.3	3858	29.1	2502	28.3	1435	28.5	0.001
Polyunsaturated fat (g/day)	2151	11.2	1080	12.2	3858	12.4	2502	13.0	1435	13.7	<0.001
Monounsaturated fat (g/day)	2151	24.5	1080	24.9	3858	24.9	2502	24.5	1435	24.9	0.73
Carbohydrates (g/day)	2151	223.7	1080	220.6	3858	222.1	2502	222.2	1435	227.5	0.23
Ever smoking during pregnancy, n, %	2020	48.7	1010	36.1	3661	26.7	2371	19.1	1405	9.3	<0.001
**Partner characteristics**											
Partner's height (cm)	1252	174.8	682	175.0	2673	176.1	1768	176.5	1135	177.6	<0.001
Paternal BMI (kg/m^2^)	1228	25.4	676	25.5	2641	25.3	1752	25.1	1132	24.6	<0.001
Partner' smoking, n,%	2076	51.1	1064	45.2	3830	39.7	2503	31.2	1459	20.1	<0.001

BMI: body mass index.

Table 2 shows the change in z-score of height per group of maternal education. A linear increase across all educational categories provided a good fit for the model and showed that for boys and girls each increase in educational level was associated with a 0.049 of a standard deviation (4.9%) increase in standardised height (95% CI: 3.7% to 6.0%). Although the magnitude of this change was greater among girls, there was no evidence of differences in z-scores of height growth by educational level between genders (interaction p-value = 0.6). All subsequent analyses were carried out jointly for boys and girls, with adjustment for gender. A 4.9% of standard deviation difference in height z-scores translates to a difference of 0.39 cm in birth length (95% CI: 0.30 cm to 0.48 cm) among children whose mothers had a degree compared to children whose mothers had no or basic education (4.9% change×2 cm (SD of birth length)×4 educational levels). Around the age of 11.5 years, the height difference between children whose mothers had a degree compared to those whose mothers had the lowest education was 1.4 cm (95% CI: 1.07 cm to 1.74 cm) (SD of height at age 11.5 = 7.26 cm).

**Table 2:** Mean differences in child's height growth (in z-score) (β) by maternal education in boys and girls.

	Boys		Girls		Combined	
Education	β	95% CI	β	95% CI	β 1	95% CI
**None/CSE**	ref	-	ref	-	ref	-
**Vocational**	0.067	−0.014, 0.147	0.047	−0.031, 0.126	0.060	0.003, 0.118
**O-level**	0.090	0.031, 0.148	0.098	0.041, 0.155	0.090	0.049, 0.132
**A-level**	0.120	0.056, 0.183	0.124	0.062, 0.187	0.124	0.078, 0.169
**Degree**	0.209	0.135, 0.283	0.230	0.158, 0.302	0.220	0.168, 0.273
**Linear trend**	0.046	0.030, 0.062	0.051	0.036, 0.067	0.049	0.037, 0.060

1 Adjusted for gender.

Table 3 shows that all potential mediating factors investigated here were associated with child's height growth (except maternal diet during pregnancy) and were therefore, potential explanatory variables of the differences of child's height growth by maternal educational in our study.

**Table 3:** Differences in child's height growth (in z-score) (β) according to several potential explanatory factors.

	β	95% CI
**Gestational age (weeks)**	0.054	0.043, 0.065
**Number of siblings**	−0.034	−0.049, −0.019
**Breast feeding**	0.067	0.035, 0.099
**Mother's age at delivery (years)**	0.011	0.008, 0.014
**Maternal height (cm)**	0.047	0.045, 0.049
**Maternal BMI (kg/m^2^)**	0.012	0.008, 0.016
**Maternal smoking**	−0.170	−0.203, −0.137
**Maternal energy intake (kcal/day)**	−0.00002	−5.5×10^−5^,7.1×10^−6^
Protein intake (g/day)	0.001	0.0002, 0.002
Total fat (g/day)	−0.0005	−0.001, 0.0001
Saturated fat (g/day)	−0.001	−0.003, −0.0002
Polyunsaturated fat (g/day)	0.002	−0.001, 0.004
Monounsaturated fat (g/day)	−0.002	−0.003, 0.0001
Carbohydrates (g/day)	−0.0003	−0.0005, −0.0001
**Paternal height (cm)**	0.041	0.039, 0.044
**Paternal smoking**	−0.048	−0.078, −0.018
**Paternal BMI** ^1^ **(kg/m^2^)**	0.018	0.012, 0.023
**Mid-parental height (cm)**	0.073	0.070, 0.076

All mean differences are adjusted for gender only.

They are not mutually adjusted for the other characteristics.

**Figure 2:**
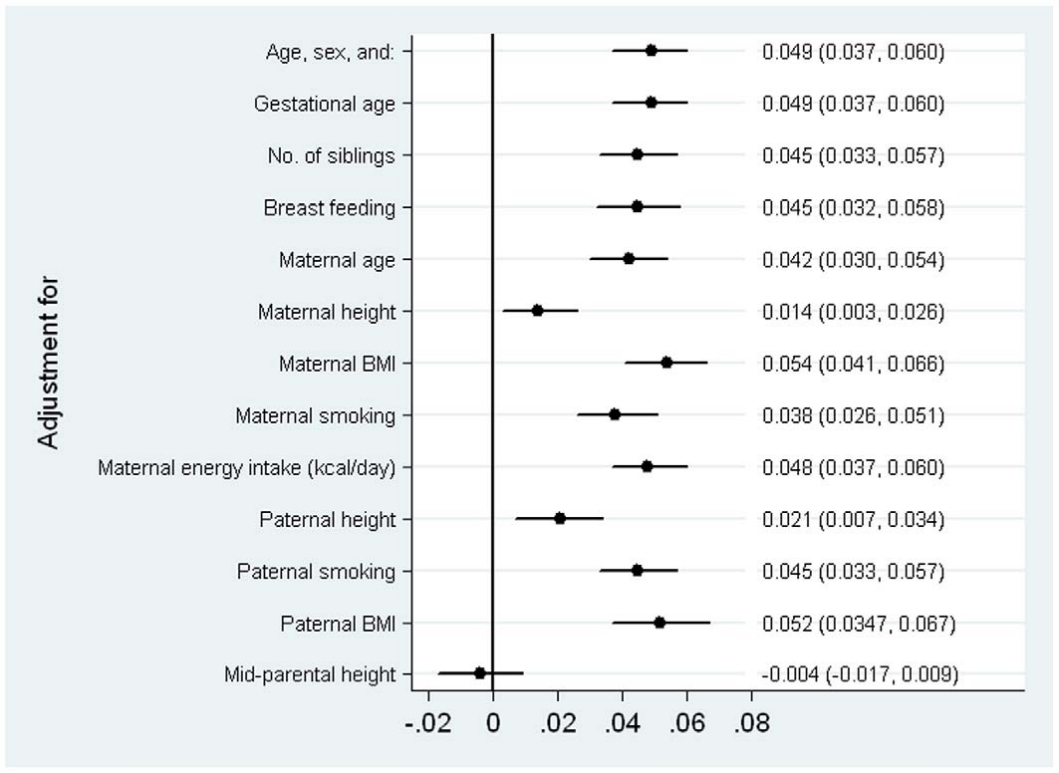
Mean difference in child's height growth (z-score) and 95% confidence interval associated with maternal education (ordinal variable) and following adjustment of potential explanatory characteristics (each coefficient presents results from a separate regression model).

Figure 2 shows the coefficient for maternal education and childhood z-scores of height growth after adjusting for each potential explanatory variable. Gestational age, number of siblings, breast feeding and mother's age at delivery did not explain the educational differences in child's height growth as shown by the negligible change in the magnitude of the association between education and height growth after adjustments. Using a finer categorization of breast feeding (Never, <1 month, 1–3 months, 3–6 months, >6 months) did not explain more of the educational inequalities in child's height (adjusted ß = 0.044 SD, 95% CI: 0.031, 0.058). Maternal smoking during pregnancy explained some of the educational inequalities. A more detailed variable indicating the number of trimesters the mother smoked (0 to 3) explained only slightly more of this association (adjusted ß = 0.036 SD, 95% CI: 0.024, 0.049). None of the specific nutrients that were analysed changed the association of maternal education with offspring height growth (results available from authors on request). Adjustment for maternal and partner's BMI slightly increased the maternal educational differences in childhood height growth. The variables that led to greater attenuation of the differences in height growth were maternal and partner's height. Adjustment for mid-parental height (combined maternal and partner's height) resulted in attenuation of the association of maternal educational with child's height growth to the null. Adjustment for all variables simultaneously, except mid-parental height, diminished but did not account for all of the maternal educational association with child's height (ß of maternal education = 0.022, 95% CI: 0.002, 0.042).

When considering growth until 10 years of age only in order to assess whether pubertal changes already occurring in some children could have influenced the results, educational inequalities in child's growth remained similar (ß = 0.048 SD, 95% CI: 0.036, 0.059). When all analyses were repeated excluding observations with standardised height values above 2 or below −2 the effect of maternal education on standardised height growth remained although the magnitude of the effect was slightly reduced (ß = 0.041 SD, 95% CI: 0.031, 0.051). Maternal and partner's height remained as the main explanatory variables of the educational differences in child's height growth (adjusted ß = −0.003; 95% CI: −0.015, 0.008), whereas adjustment for all other characteristics, but not mid-parental height, resulted in attenuation but some association remained (adjusted ß = 0.018, 95% CI: 0.002, 0.033). Finally, educational inequalities in child's growth were slightly greater after removing those with only one or all ten height measures (ß = 0.051 SD, 95% CI: 0.039, 0.064) and the role of the explanatory variables remained the same (maternal education ß adjusted for mid-parental height = 0.0003; 95% CI: −0.014, 0.014; maternal education ß adjusted for all other characteristics except mid-parental height = 0.026; 95% CI: 0.007, 0.014).

## Discussion

Among children born in the UK in the early 1990s, those born to mothers with higher educational levels were taller than those born to mothers of lower educational levels. These height inequalities were present at birth and persisted over time (0.39 cm in birth length, 1.4 cm at the age of 11.5 years). Mid-parental height fully explained the differentials in child's height growth across maternal educational levels. Although most of the other explanatory variables investigated were associated with the child's height and were socially patterned, they accounted for little of the maternal educational inequalities in the child's height.

The improvements in pre-natal and maternal care and child nutrition, along with fewer childhood infections would suggest that inequalities in height due to environmental exposures should have decreased or disappeared in high income countries [17], [27]. However, this and previous reports from the ALSPAC cohort [18], [19], as well as from other cohorts from high-income countries [28]–[30] still find height inequalities in contemporary children. Several studies have sought to identify the factors that explain these differences and their relative importance in explaining the inequalities.

The Boyd Orr study, a cohort of 4999 children surveyed between 1937–39 in the UK, showed a general pattern of greater stature and body proportions (leg and foot length, trunk and shoulder width were also investigated) with better childhood socioeconomic and housing circumstances as well as diet [31]. Parents' height was available for a sub-sample of participants in this study but it did not explain the association between child's height and socioeconomic circumstances [31]. Two birth cohorts 40 years apart (1947 and 1987) in Newcastle showed a similar 4 cm difference in height between the two extreme deprivation groups in both cohorts; mid-parental height explained half of that difference (i.e. attenuated the point estimate by 50%) [28]. The lack of expected reduction in the socioeconomic height differences, between the two cohorts might be due to using different indicators of SEP to report these differences [32]. Rona et al found between 1.0 and 2.2 cm differences in height across different ages (children aged 5 to 11.5 years) between children whose fathers had a manual occupation compared to non-manual occupations [33]. In this study, mother and father's height was the variable with the strongest association with child's height although it did not explain all of the socioeconomic differentials in height [34]. The NHANES III study in the US, found differences in childhood height at ages 6 to 16 between race/ethnicity and by household size but not with parental education or income [29]. Racial inequalities may have included socioeconomic inequalities not captured by education or income. In Sweden, Lindgren reported no differences in average height according to SEP, indexed with father's social class and single parent families, in a sample of school girls and boys aged 10 to 16 (born in 1954/55) [35]. However, other studies have reported differences associated with occupational and income levels in adult height in later cohorts [30]. Finally, Li et al compared changes in height inequalities between two generations, the 1958 birth cohort and that of their offspring [17]. They found that inequalities in height narrowed from 2–3 cm at the age of 7 in the first generation to less than 1 cm in their children, born about 26 years later. Greater increases in height among the manual classes in the offspring generation explained the diminished inequalities. In addition, pre- and post-natal exposures explained less variance in the younger generation, giving support to claims that the effect of environmental determinants might be lessening in high income countries [36]. Conversely, a recent study in a contemporary cohort from Belarus, a country undergoing major social and economic change, found that the magnitude of inequalities in parents was the same as that in the offspring, and that mid-parental height explained some, but not all, of the inequalities [37].

We found a similar pattern of association of potentially modifiable exposures with height growth in ALSPAC with those reported in the literature [5], [14], [38]. Namely, greater gestational age and older mothers, breast feeding, and greater maternal and paternal BMI were associated with increased stature in children whereas having more siblings and maternal and paternal smoking were associated with reduced stature. Some of these associations were confounded by maternal educational differences. For example, breastfeeding was no longer associated with child's height after accounting for maternal education (β = 0.024, 95% CI: −0.010, 0.058). In the 1958 UK birth cohort the positive association between breastfeeding and offspring's height was substantially weakened after adjustment for parental height, early life exposures and parental social class [39]. Maternal nutrition during pregnancy was not related to child's height.

However, although these factors were associated with child's height they explained little of the inequalities, whereas educational inequalities in childhood height were no longer present when comparing children whose parents had the same mid-parental height. Li et al compared the effect of parental height in the two generations and found parental height had a stronger association in the offspring than the parents' generation [36]. Thus, it would appear that factors related to parental height are the main driver of inequalities in child's height growth in the contemporary UK population.

Parental height is often used as proxy for the genetic component of height. Indeed, the fact that the combination of both parents' height was necessary to account for the height differences, rather than maternal height alone, does seem to point to a strong genetic contribution. However, parental height partly reflects the embodiment of a range of social and environmental characteristics shared within a family that relate to the parents' stature and will also influence offspring height (e.g. socially patterned behaviours that are transmitted through generations and therefore can influence parental height as well as that of the offspring). These could be transmitted to subsequent generations through several mechanisms. Assortative mating by social class can result in parents of higher education, who are taller, transmitting their genetic, social and environmental characteristics to their children who then also grow to be taller. In the ALSPAC cohort, maternal and partner's height increased with increasing education and the educational level of the mother's partner tended to be similar to her own (65% of the mothers with no or basic education had partner with no or basic education whereas 75% of mothers with a degree had a partner with a degree). On the other hand, a gene-by-environment correlation with respect to genetic variants related to height could also generate this pattern. Mid-parental height might also incorporate effects due to exposures that are not measured accurately, e.g. maternal smoking during pregnancy. Smoking has a negative effect on height [14] and it has a negative association with educational level in this population. The strong pressure on women to give up smoking during pregnancy is likely to result in them underreporting their true exposure. Thus, the unmeasured effect of smoking may, in part, be incorporated into mid-parental height.

The next step in understanding the associations of height and height inequalities in high income countries requires understanding the determinants of parental height that are transmitted across generations, and disentangling the different aspects, genetic and environmental, that are captured by this variable. On the one hand, we need more knowledge on the genetic variants related to height, as up until now these can only explain about 45% of the height variance [40]. On the other hand, other study designs including twins, siblings, paternal versus maternal characteristics and transmission of intergenerational effects will offer additional insight into the role of genetic versus environmental exposures in explaining current inequalities in height.

Some methodological limitations need to be considered in interpreting results from this study. Birth measures were available for about 60% of the total sample. Height measures from the first child visit were available for more than 80% of the cohort but this decreased to about 50% by age 11.5 years. There were fewer height measurements for children of mothers with lower levels of education. This loss to follow-up will only bias the results if the direction of the association in those who did not participate or were lost to follow-up was in a different direction to the one reported here. A previous report from this cohort analysed participants with at least 9 height measures and found similar results as to when children with 1 or more measures were included [18]. We might, however, have underestimated the true differences of child height according to maternal education, if those children who did not participate or were lost to follow up had mothers with lower education and were shorter than the ones who remained in the study. Thus, it is possible that the magnitude of inequalities in height presented here are the lower estimate of what could be found in the whole cohort.

As some heights were standardised over a wider age range (intervals were collapsed when there were too few observations for appropriate calculation of a z-score) this resulted in a positive correlation of height z-scores with age, and therefore all models were additionally adjusted for an age z-score to fully account for differential ages at measurement. The effect of education on height did not differ between the models that included this additional adjustment and those that didn't.

In conclusion, inequalities in child's growth, although relatively small in magnitude, persist in England. These were fully explained by maternal and paternal reported height. Disentangling the genetic and environmental factors that this variable captures will help understanding the preventable factors that underlie height inequalities in rich income countries.

## Supporting Information

Appendix S1 **Calculation of mid-parental height.**
(DOCX)

## References

[1] Perola M, Sammalisto S, Hiekkalinna T, Martin NG, Visscher PM (2007). Combined genome scans for body stature in 6,602 European twins: evidence for common Caucasian loci.. PLoS Genet.

[2] Subramanian SV, Ozaltin E, Finlay JE (2011). Height of nations: a socioeconomic analysis of cohort differences and patterns among women in 54 low- to middle-income countries.. PLoS ONE.

[3] Cole TJ (2000). Secular trends in growth.. Proc Nutr Soc.

[4] Kac G (1999). Secular height trend: a literature review.. Cad Saude Publica.

[5] Rona RJ, Chinn S (1995). Genetic and environmental influences on growth.. J Med Screening.

[6] Barker DJP, Robinson RJ (1993). Fetal and infant origins of adult disease.

[7] Ravelli GP, Stein ZA, Susser MW (1976). Obesity in young men after famine exposure in utero and early infancy.. N Engl J Med.

[8] Susser M, Stein Z (1994). Timing in prenatal nutrition: a reprise of the Dutch Famine Study.. Nutr Rev.

[9] McCormack VA, dos Santos Silva I, De Stavola BL, Mohsen R, Leon DA (2003). Fetal growth and subsequent risk of breast cancer: results from long term follow up of Swedish cohort.. BMJ.

[10] Kuh D, Wadsworth M (1989). Parental height: childhood environment and subsequent adult height in a national birth cohort.. Int J Epidemiol.

[11] Baker IA, Elwood PC, Hughes J, Jones M, Moore F (1980). A randomised controlled trial of the effect of the provision of free school milk on the growth of children.. J Epidemiol Community Health.

[12] Elwood PC, Sweetnam PM, Gray OP, Davies DP, Wood PD (1987). Growth of children from 0–5 years: with special reference to mother's smoking in pregnancy.. Ann Hum Biol.

[13] Meredith HV (1975). Relation between tobacco smoking of pregnant women and body size of their progeny: a compilation and synthesis of published studies.. Hum Biol.

[14] Leary S, Davey Smith G, Ness A (2006). Smoking during pregnancy and the components of stature in offspring.. Am J Hum Biol.

[15] Bernstein IM, Plociennik K, Stahle S, Badger GJ, Secker-Walker R (2000). Impact of maternal cigarette smoking on fetal growth and body composition.. Am J Obstet Gynecol.

[16] Zaren B, Lindmark G, Bakketeig L (2000). Maternal smoking affects fetal growth more in the male fetus.. Paediatr Perinat Epidemiol.

[17] Li L, Manor O, Power C (2004). Are inequalities in height narrowing? Comparing effects of social class on height in two generations.. Arch Dis Child.

[18] Howe LD, Tilling K, Galobardes B, Smith GD, Gunnell D (2010). Socioeconomic differences in childhood growth trajectories: at what age do height inequalities emerge?. Journal of Epidemiology and Community Health.

[19] Clark EM, Ness A, Tobias JH (2005). Social Position Affects Bone Mass in Childhood Through Opposing Actions on Height and Weight.. Journal of Bone and Mineral Research.

[20] Steckel RH (1995). Stature and the standard of living.. J Economic Literature.

[21] Batty GD, Shipley MJ, Gunnell D, Huxley R, Kivimaki M (2009). Height, wealth, and health: An overview with new data from three longitudinal studies.. Economics & Human Biology.

[22] Gunnell D, Okasha M, Davey Smith G, Oliver SE, Sandhu J (2001). Height, leg length, and cancer risk: a systematic review.. Epidemiologic Reviews.

[23] Golding J, Pembrey M, Jones R, The Alspac Study Team (2001). ALSPAC-The Avon Longitudinal Study of Parents and Children.. Paediatric and Perinatal Epidemiology.

[24] Galton F (1889). Natural Inheritance.

[25] Laughlin HH (1935). How to use the specific formula of heredity.. PNAS.

[26] Howe LD, Tilling K, Lawlor DA (2009). Accuracy of height and weight data from child health records.. Arch Dis Child.

[27] Silventoinen K, Kaprio J, Lahelma E, Koskenvuo M (2000). Relative effect of genetic and environmental factors on body height: differences accross birth cohorts among Finish men and women.. Am J Public Health.

[28] Wright CM, Parker L (2004). Forty years on: the effect of deprivation on growth in two Newcastle birth cohorts.. Int J Epidemiol.

[29] Dowd JB, Zajacova A, Aiello A (2009). Early origins of health disparities: burden of infection, health, and socioeconomic status in U.S. children.. Soc Sci Med.

[30] Silventoinen K, Lahelma E, Lundberg O, Rahkonen O (2001). Body height, birth cohort and social background in Finland and Sweden.. Eur J Public Health.

[31] Whitley E, Gunnell D, Davey Smith G, Holly JMP, Martin RM (2008). Childhood circumstances and anthropometry: The Boyd Orr cohort.. Annals of Human Biology.

[32] Chinn S, Rona RJ (2004). Commentary: The relation of growth to socioeconomic deprivation.. Int J Epidemiol.

[33] Rona RJ, Swan AV, Altman DG (1978). Social factors and height of primary schoolchildren in England and Scotland.. J Epidemiol Community Health.

[34] Rona RJ, Swan AV, Altman DG (1978). Social factors and height of primary schoolchildren in England and Scotland.. Journal of Epidemiology and Community Health.

[35] Lindgren G (1976). Height, weight and menarche in Swedish urban school children in relation to socio-economic and regional factors.. Ann Hum Biol.

[36] Li L, Power C (2004). Influences on childhood height: comparing two generations in the 1958 British birth cohort.. Int J Epidemiol.

[37] Patel R, Lawlor DA, Kramer MS, Davey Smith G, Bogdanovich N (2011). Socioeconomic inequalities in height, leg length and trunk length among children aged 6.5 years and their parents from the Republic of Belarus: Evidence from the Promotion of Breastfeeding Intervention Trial (PROBIT).. Annals of Human Biology.

[38] Goldstein H (1971). Factors influencing the height of seven year old children–results from the National Child Development Study.. Hum Biol.

[39] Li L, Manor O, Power C (2004). Early environment and child-to-adult growth trajectories in the 1958 British birth cohort.. Am J Clin Nutr.

[40] Yang J, Benyamin B, McEvoy BP, Gordon S, Henders AK (2010). Common SNPs explain a large proportion of the heritability for human height.. Nat Genet.

