# Supplementary material for: Social Inequalities in Height: Persisting Differences Today Depend upon Height of the Parents
Source: PLoS One. 2012 Jan 6;7(1):e29118. doi: 10.1371/journal.pone.0029118 (PMC3253075; doi:10.1371/journal.pone.0029118)
Supplement: Appendix S1 — Calculation of mid-parental height. (DOCX) [file pone.0029118.s001.docx]

Appendix S1. Calculation of mid-parental height

Mid-parental height was calculated using an adaptation of Galton’s formula [24,25]. The original formula was:

Mid-parental height = (father’s adult height + mother’s adult height x 1.08) / 2

The correction factor of 1.08 in this formula was based on the ratio of average fathers height to the average mothers height in Galton’s population. We calculated this factor for the Alspac sample included in this analysis:

Correction factor in Alspac = 176.0 / 164.0 = 1.07

Thus, mid-parental’s height in the Alspac was calculated as follows:

Mid-parental height = (father’s adult height + mother’s adult height x 1.07) / 2
